# Antimicrobial Susceptibility Testing of Metronidazole and Clindamycin against *Gardnerella vaginalis* in Planktonic and Biofilm Formation

**DOI:** 10.1155/2020/1361825

**Published:** 2020-06-16

**Authors:** Ting Li, Zhan Zhang, Fengjuan Wang, Yuanhui He, Xiaonan Zong, Huihui Bai, Zhaohui Liu

**Affiliations:** ^1^Department of Gynecology, Beijing Obstetrics and Gynecology Hospital, Capital Medical University, Beijing 100026, China; ^2^Department of Obstetrics, Beijing Obstetrics and Gynecology Hospital, Capital Medical University, Beijing 100026, China; ^3^Department of Obstetrics and Gynecology, Peking University First Hospital, Beijing 100034, China; ^4^Department of Microecological Laboratory, Beijing Obstetrics and Gynecology Hospital, Capital Medical University, Beijing 100026, China

## Abstract

**Background:**

Bacterial vaginosis (BV), one of the most common vaginal ecosystem-related microbiologic syndromes, is the most common disorder in women of reproductive age. *Gardnerella* (*G.*) *vaginalis* is the predominant species causing this infection. Our aim was to compare the antimicrobial susceptibilities of metronidazole and clindamycin against *G. vaginalis* at planktonic and biofilm levels.

**Methods:**

From September 2019 to October 2019, we recruited a total of 10 patients with BV who underwent gynecological examinations at Beijing Obstetrics and Gynecology Hospital. *G. vaginalis* isolates were obtained from the vagina and identified using their characteristic colony morphology. Sequence data of clinical *G. vaginalis* isolates were confirmed by comparing 16S rDNA sequences. Subsequently, clinical isolates were evaluated for antimicrobial susceptibilities *in vitro* to metronidazole and clindamycin at planktonic and biofilm levels. The minimum inhibitory concentration (MIC) for metronidazole and clindamycin was evaluated by antimicrobial susceptibility testing. The minimum biofilm eradication concentration (MBEC) was evaluated by the biofilm inhibition assay.

**Results:**

Planktonic clinical isolates showed a significantly higher susceptibility rate (76.67%) and lower resistance rate (23.33%) to clindamycin than to metronidazole (susceptibility rate: 38.24%; resistance rate: 58.82%; *P* < 0.05 for both). Furthermore, in comparison to planktonic isolates, the minimum inhibitory concentration (MIC) of metronidazole was significantly higher for biofilm-forming isolates (7.3 ± 2.6 *μ*g/mL vs. 72.4 ± 18.3 *μ*g/mL; *P*=0.005); the resistance rate was 27.3%, and the minimum biofilm eradication concentration (MBEC) was >128 *μ*g/mL. Moreover, the MIC of clindamycin was higher too for biofilm-forming isolates (0.099 ± 0.041 *μ*g/mL vs. 23.7 ± 9.49 *μ*g/mL; *P*=0.034); the resistance rate was 27.3%, and the MBEC of clindamycin was 28.4 ± 6.50 *μ*g/mL.

**Conclusion:**

Our results indicate that in comparison to metronidazole, clindamycin seems to be a better choice to tackle *G. vaginalis* as it exhibits a relatively higher susceptibility rate and lower resistance rate.

## 1. Introduction

Bacterial vaginosis (BV) is one of the most common disorders of the lower genital tract in women of childbearing age. It represents an abnormal vaginal ecosystem, characterized by an initial decrease of healthy *Lactobacillus*‐dominated vaginal microbiota and a subsequent increase of anaerobic and facultative bacteria such as *Gardnerella* (*G.*) *vaginalis*, *Atopobium vaginae*, and *Prevotella bivia* [1]. *G. vaginalis* has been isolated in up to 95% cases, and it is the most typical and widely studied pathogen associated with BV [2]; the prevalence varies depending on race and ethnicity. For example, the National Health and Nutrition Examination Survey 2001–2004 reported that the prevalence of BV was 29.2% in women of reproductive age in the United States, and only 15.7% women with BV reported vaginal symptoms [3]. Furthermore, in a study conducted by Zhang et al. [4] on 1,218 married women in China, BV was the second most common disorder with an estimated prevalence of 10%; in the Tibetan area of Sichuan Province, China, the prevalence of BV was reported to be 51.6% in a study conducted by Dai et al. [5]. BV can be considered a biofilm-associated infection, with a dense-structured polymicrobial biofilm consisting primarily of *G. vaginalis* adhering to the vaginal epithelium [6]. This biofilm sometimes ascends to the upper genital tract forming a polymicrobial endometrial and fallopian tube biofilm [7], and it may explain the associated with an increased risk of developing pelvic inflammatory diseases and adverse pregnancy outcomes, such as premature birth, abortion, and chorioamnionitis [1]. Moreover, BV is also associated with an increased risk of acquisition of sexually transmitted infections, such as human immunodeficiency virus [8] and *Chlamydia trachomatis* infection [9], and it may play a role as cofactors in human papillomavirus-mediated cervical carcinogenesis [10]. The endometrial cavity is not sterile in most women with the presence of low levels of bacteria in the uterus, which is not associated with clinically significant inflammation [11]. However, the exact role of biofilm in relation to infectious diseases of the upper genital tract remains uncertain [12].

As the first-line of therapy for BV, the Centers for Disease Control and Prevention (CDC) recommends the use of oral or vaginally applied metronidazole or clindamycin [13]. Metronidazole, a derivative of nitroimidazole, is widely used. It may be administered orally at 500 mg twice a day for 7 days or applied intravaginally in the form of a 0.75% gel once a day for 5 days [1]. Intravaginal application of clindamycin at bedtime for 7 days is also a common treatment regimen, but relapse can reportedly occur [1]. The relapse and recurrence of BV are the biggest challenges to current therapies; the recurrence rate is >50% mainly because of the development of a multispecies biofilm, with *G. vaginalis* being one of the dominant species [14]. Nevertheless, both metronidazole and clindamycin have been reported to achieve cure rates of 70%–96%, with recurrence rates of 49%–66%; the relapse rates of metronidazole or clindamycin are quite high at 67% within 6–12 months [15]. The precise relationship between BV-associated biofilm-forming bacteria and treatment failure, however, still remains unknown. Accordingly, disrupting *G. vaginalis* biofilms seems to be a promising step toward developing a more sustainable way to treat BV and tackle its recurrence [16].

Many studies have reported the antimicrobial action of metronidazole and clindamycin on *G. vaginalis*, which is still considered to be the predominant bacteria causing BV. In the present *in vitro* study, we investigated the susceptibilities of planktonic *G. vaginalis* and biofilms to metronidazole and clindamycin and compared the antimicrobial abilities of these two antibiotics.

## 2. Methods

### 2.1. Ethical Approval of the Study Protocol

This study was approved by the Ethics Committee of Beijing Obstetrics and Gynecology Hospital, Beijing, China (2019-KY-030-01), and it was conducted in accordance with the principles expressed in the Declaration of Helsinki. All eligible participants provided written informed consent to be included in this study.

### 2.2. Patients and Sample Collection

From September 2019 to October 2019, we recruited a total of 10 patients with BV who underwent gynecological examinations at Beijing Obstetrics and Gynecology Hospital. The exclusion criteria included pregnancy, menstruation, sexual intercourse, or application of any intravaginal product within the last 24 h, use of antibiotics in the last month, and lower genital tract malignancy. We also excluded women who had previously undergone cervical surgery or pelvic radiation therapy; those with Trichomonas vaginitis, vulvovaginal candidiasis, aerobic vaginitis, and other vaginal infections; and those allergic to metronidazole or clindamycin. Patients aged 18–50 years and with a Nugent score of ≥7 were included.

Regular gynecological examinations were performed, and vaginal secretions of patients with BV were sampled from the upper third of the vaginal wall using aseptic endocervical cotton swabs. We stained the smears using Gram's method; subsequently, the smears were examined by one observer at ×400 magnification in an optical microscope (Leica Microsystems, Mannheim, Germany), and a Nugent score was assigned to each sample. Smears with a Nugent score of ≥7 were considered to be diagnostic of BV, while those with a score of 4–6 were diagnostic of intermediate BV, and 0–3 were interpreted as normal flora [17].

### 2.3. Antimicrobial Preparation

Metronidazole was purchased from Fuzhou Haiwang Fuyao Pharmaceutical Co., Ltd (Fujian, China), and clindamycin palmitate was purchased from Guangzhou Yipinhong Pharmaceutical Co., Ltd (Guangdong, China). Metronidazole (0.0625–64 *µ*g/mL) and clindamycin (0.03125–32 *µ*g/mL) were stored as stock solutions, and to attain the desirable concentration, they were serially diluted using modified brain heart infusion (BHI) broth (Difco, Sparks, MD).

### 2.4. Identification and Isolation of *G. vaginalis*

10 clinical isolates of *G. vaginalis* were identified using their characteristic colony morphology; representative colonies were selected from each vaginal secretion. We used Columbia blood agar base (Sigma-Aldrich, US), and colonies were isolated to purity. Gram stain showed that the isolates were Gram-variable pleomorphic rods, and the catalase reaction was negative. DNA from clinical *G. vaginalis* isolates and *A. vaginae* isolates was extracted using the QIAamp DNA Mini Kit (QIAGEN, Germany). For identifying the isolates, 16S rRNA gene hypervariable V1–V3 region was amplified using the primers 27F (5′-AGAGTTTGATCCTGGCTCAG-3′) and 1492R (5′-GGTTACCTTGTTAGACTT-3′). Sequence data of clinical *G. vaginalis* isolates were confirmed by comparing 16S rDNA sequences to the GenBank data library using the advanced gapped BLAST program. The purified isolates were stored at −80°C in the De Man, Rogosa, and Sharpe (MRS) broth containing 30% glycerol.

### 2.5. *G. vaginalis* and Planktonic Cultures

The clinical isolates were cultured using Columbia blood agar base (Becton Dickinson, Rockville, MD) at 37°C under anaerobic conditions in an anaerobic glove box (Coy Laboratory Products, Inc., Grass Lake, MI); the anaerobic chamber contained 5% H_2_, 5% CO_2_, and 90% N_2_. Different isolates were plated onto the agar medium and then suspended in modified BHI broth using a 0.5 McFarland standard, for a total concentration of 1.5 × 10^8^ CFU/mL, as previously described [18]. *G. vaginalis* ATCC®14018 was used as a control for the tests carried out under anaerobic conditions.

### 2.6. Biofilm Formation and Quantification

To develop the biofilm model of *G. vaginalis*, a starting inoculum of approximately 10^6^ CFU/mL of prepared bacterial suspension in the BHI broth with 0.4% (w/v) glucose was added to different concentrations of metronidazole and clindamycin and inoculated into a 96-well microplate (Falcon, Corning Inc., Corning, NY) for 48 h at 37°C, 5% CO_2_. The BHI media and biofilm culture without any compounds served as controls. *Bacteroides fragilis* ATCC®14018 was used as a quality control bacterium and cultivated under anaerobic conditions. The plates were incubated for 48 h, and the growth medium was replaced every 24 h. Crystal violet staining (Sigma-Aldrich, St. Louis, MO, US) was used to quantify the total amount of biofilm biomass [19]. Biofilms were then analyzed after a total of 48 h of incubation at an optical density of 595 nm (OD_595_) using a microplate reader (Bio-Rad Laboratories, Hercules, CA, USA). The isolates were classified into four categories on the basis of their ability to form biofilms according to the cut-off OD value (ODc), which was defined as three standard deviations (SD) above the mean OD of the negative control [20]: OD ≤ ODc, no biofilm producer; OD ≤ 2 × ODc, weak biofilm producer; OD ≤ 4 × ODc, moderate biofilm producer; and 4 × ODc < OD, strong biofilm producer.

### 2.7. Antimicrobial Susceptibility Testing

Clinical *G. vaginalis* isolates were evaluated for antimicrobial susceptibilities *in vitro* to metronidazole and clindamycin (Sigma-Aldrich, St. Louis, MO, US) targeting planktonic cells; to achieve this, we used the anaerobic agar dilution method described by the Clinical and Laboratory Standards Institute (CLSI).

One-hundred microliters of the prepared bacterial suspension (10^6^ CFU/mL) was added to different concentrations of metronidazole and clindamycin at 37°C. After 48 h, the bacterial growth was evaluated by taking an endpoint reading at OD_595_ with a microplate reader (Bio-Rad Laboratories, Hercules, CA, USA). The minimum inhibitory concentration (MIC) for metronidazole and clindamycin was defined as the lowest antibiotic concentration yielding marked reduction in the growth or no growth at all. Under the same conditions, *G. vaginalis* ATCC®14018 was tested using the broth microdilution assay. The microbiological susceptibility and resistant breakpoints for metronidazole (<8 *μ*g/mL and ≥32 *μ*g/mL) and clindamycin (<2 *μ*g/mL and ≥8 *μ*g/mL), as defined by CLSI, were used for interpreting MIC results [21].

### 2.8. Biofilm Inhibition Assay

Biofilms were used to evaluate the activity of metronidazole and clindamycin. After 48 h of incubation with antimicrobial-containing medium, biofilms were washed twice with sterile phosphate-buffered saline, dried, and stained with 0.2% crystal violet (Sigma-Aldrich); and the biofilms were solubilized in 100 *μ*L of 95% ethanol for 5 min. The absorbance of the stain was measured to quantify the cell viability of each biofilm [18]. The minimum biofilm eradication concentration (MBEC) was defined as the lowest concentration of an antibiotic that completely inhibited the growth of microorganisms, indicating complete biofilm eradication, as previously described [22].

### 2.9. Statistical Analysis

Statistical Package for the Social Sciences v13.0 (SPSS Inc., USA) was used for statistical analysis. Results are presented as mean ± SD of the mean of at least triplicates. The main test used was the *χ*^2^ test; *P* ≤ 0.05 indicated statistical significance.

## 3. Results

**Figure 1:**
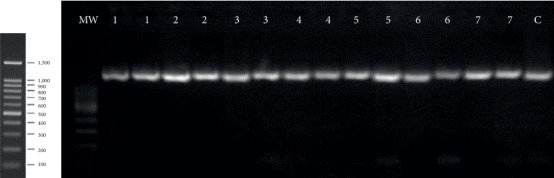
Representative electropherograms of *Gardnerella vaginalis* PCR identification. MW: molecular weight standard; lanes 1–7 in duplicate: bacteria isolated from vaginal secretions; *Gardnerella vaginalis* ATCC®14018 was the standard strain used as a quality control bacterium (C). Expected amplicon size: 1400 bp.

During the study period, a total of 10 isolates of *G. vaginalis* were isolated. Clinical information for the patients was obtained as a routine laboratory practice in the study. The identity of all presumptively isolated *G. vaginalis* was confirmed by the PCR (Figure 1). The study population had a mean age of 37.7 ± 11.03 years, mean Nugent scores of 7.60 ± 1.71, and mean pH values of 4.69 ± 0.40. Five (50.0%) of these were with abnormal vaginal discharge.

Biofilm formation by individual isolates was heterogeneous. 10 clinical *G. vaginalis* isolates and 1 *G. vaginalis* isolate (ATCC®14018) could form biofilms: 5 (45.5%) of these were weak biofilm producers, 5 (45.5%) were moderate biofilm producers, and 1 (9.0%) was a strong biofilm producer (Table 1).

**Figure 2:**
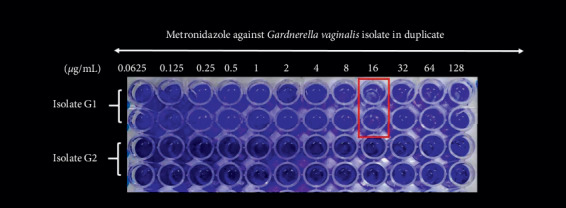
Biofilm formation testing by one clinical isolates of *Gardnerella vaginalis* (isolate nos. G1 and G2) against metronidazole. Clinical *Gardnerella vaginalis* (isolate nos. G1 and G2) was evaluated for antimicrobial susceptibilities in vitro to metronidazole targeting the biofilm. To develop the biofilm model, a starting inoculum of approximately 10^6^ CFU/mL of prepared bacterial suspension in the BHI broth with 0.4% (w/v) glucose was added to different concentrations of metronidazole and inoculated into a 96-well microplate for 48 h at 37°C, 5% CO_2_. Crystal violet staining was used to quantify the total amount of biofilm biomass. Light blue wells with red box were considered as wells without growth, the minimum biofilm eradication concentration (MBEC), defined as the lowest concentration of an antibiotic that completely inhibited the growth of microorganisms, indicating complete biofilm eradication (No. G1 : MBEC  = 16 *μ*g/mL; No. G2 : MBEC  > 128 *μ*g/mL).

In comparison to planktonic clinical isolates, the MIC of metronidazole was significantly higher for biofilm-forming isolates (7.3 ± 2.6 *μ*g/mL vs. 72.4 ± 18.3 *μ*g/mL; *P*=0.005; Table 1 and Figure 2), indicating a significant correlation between resistance to metronidazole and the ability of *G. vaginalis* isolates to form biofilms. Six of the 11 biofilm-forming isolates showed a high resistance to metronidazole, with a resistance rate of 54.5%; MBEC was >128 *μ*g/mL. On the basis of these results, the MBEC was not achieved with either metronidazole or clindamycin even at their highest concentrations. There was no significant linear correlation between the ability to form biofilms and the ratio of MIC values (i.e., ratio of the MIC value with and without biofilm formation; mean value 4.0 *μ*g/mL, range 2.0–16.0 *μ*g/mL, *P*=0.390).

As for clindamycin as shown in Table 1, the MIC of clindamycin was higher too for biofilm-forming isolates (0.099 ± 0.041 *μ*g/mL vs. 23.7 ± 9.49 *μ*g/mL; *P*=0.034); the resistance rate was 27.3% (3/11). Unlike metronidazole, there was a significant linear correlation between the ability to form biofilms and the ratio of MIC values (i.e., ratio of the MIC value with and without biofilm formation; mean value 16.0 *μ*g/mL, range 8.0–2048.0 *μ*g/mL, *P*=0.025, and correlation coefficient = 0.668). The MBEC of clindamycin was 28.4 ± 6.50 *μ*g/mL.

Our results indicated a significant difference between the MIC ratio of the two antibiotics with and without biofilm formation (*P*=0.083). The results for *G. vaginalis* showed that the tube MBEC/MIC ratio against metronidazole varied from 0.25 to 2048. The MBEC/MIC ratio against clindamycin varied from 64 to 1025, indicating significant differences in the effectiveness of two antibiotics in eradicating a biofilm population of two organisms.

## 4. Discussion

The BV recommended treatments are metronidazole and clindamycin, by the guidelines from the CDC and the American College of Obstetricians and Gynecologists (ACOG) [13, 23]. However, the recommended treatments are still unsatisfactory, and high recurrence rates and resistance rates are frequent because the pathogenesis process involving *G. vaginalis* is not yet well understood. Biofilms are protective for the bacteria residing within as they can trap antibiotics before they reach their target [24], protect bacteria from the effects of the host immune system [25], and keep bacteria in a metabolically quiescent state induced by nutrient limitation inside the biofilm [26]. The central role of *G. vaginalis* in BV is attributed to its higher virulence potential [27] and ability to form biofilms that are stronger than those formed by other BV-associated bacteria [28, 29]. Thus, *G. vaginalis* remains the primary pathogen of interest to study BV occurrence and recurrence. Efforts have been made to explore which microorganisms participate in the formation of a polymicrobial BV biofilm. In this study, we tested the ability of 10 clinical isolates of *G. vaginalis* to form biofilms; most of them were noted to be weak or moderate producers of biofilms *in vitro*.

CDC recommends that all symptomatic BV women should be treated using metronidazole and/or clindamycin [13]. Metronidazole, a first-generation nitroimidazole, is effective against anaerobes, but it has little or no activity against aerobes. BV-associated bacteria such as *G. vaginalis* is also sensitive to the hydroxymetabolite of metronidazole, which reportedly has a stronger antibiotic activity than the parent compound [30]. The metronidazole therapy is associated with several adverse effects such as nausea, vomiting, and gastrointestinal complaints [31]. Clindamycin, as a lincosamide, has anti-inflammatory properties with a broader range of activity against BV than metronidazole [32]. A previous study demonstrated that BV is a polymicrobial disorder that harbors particularly taxon-rich and diverse bacterial communities, which include a combination of not only anaerobic microorganisms but also other abnormal subtypes of mixed microorganisms found in women from China [33]. BV subtypes dominated by anaerobes can be more successfully treated using metronidazole, whereas clindamycin may be active against both metronidazole-sensitive and other BV-associated strains with different microbial communities.

In a previous study, *G. vaginalis* isolates were reported to be susceptible to metronidazole and clindamycin with similar MIC values [34]. In this *in vitro* study, clindamycin showed a significantly higher susceptibility rate and lower resistance rate against planktonic clinical isolates than those shown by metronidazole. Compared to clindamycin, the susceptibility rate of clinical *G. vaginalis* isolates to metronidazole was significantly lower (*P*=0.003). We found that biofilm's antibiotic administration equals to ∼2048x the MIC. Moreover, we found *G. vaginalis* biofilms to be highly tolerant to both metronidazole and clindamycin; this may create clinical challenges for obvious reasons. Surprisingly, a microbiologically significant reduction of biofilms could not be achieved with a clinically acceptable concentration of metronidazole for all biofilm-forming isolates, even for those with a weak biofilm-forming ability. With regard to treatment with clindamycin alone, coadministration of *Lactobacillus* preparations is recommended to help in the restoration of vaginal microenvironment. It is safe to assume that the high recurrence rate of BV is associated, at least in part, with the biofilm-formation ability of *G. vaginalis*. Due to the limited survey samples, a long-term and further study should be conducted to help us understand coculturing biofilm with *Lactobacillus* comprehensively and objectively, warranted to identify novel therapeutics that target vaginal biofilms in order to tackle both the occurrence and recurrence of BV.

In summary, in comparison to metronidazole, clindamycin seems to be a better choice to tackle *G. vaginalis*; as according to our results, clindamycin showed a rather higher susceptibility rate and lower resistance rate at planktonic and biofilm levels. The recurrence of BV and antibiotic tolerance of BV-associated microorganisms may be associated with the ability of *G. vaginalis* isolates to form biofilms. Clinical *G. vaginalis* isolates that form biofilms can be hard to eradicate; therefore, further studies should be conducted with the aim of exploring new therapies that can be used in combination with currently known antibiotics, without affecting the normal vaginal flora, in order to prevent the formation of new biofilms and to eradicate the presence of persistent biofilms in patients with BV.

## Figures and Tables

**Table 1:** Minimum inhibitory concentration (MIC) and minimum biofilm eradication concentrations (MBEC) for *Gardnerella vaginalis* at the planktonic and biofilm levels.

Isolate no.	Biofilm producer	Metronidazole				Clindamycin			
		Planktonic MIC (*μ*g/mL)	Biofilm MIC (*μ*g/mL)	MIC ratio^a^	MBEC (*μ*g/mL)	Planktonic MIC (*μ*g/mL)	Biofilm MIC (*μ*g/mL)	MIC ratio^a^	MBEC (*μ*g/mL)
G1	Moderate	8	16	2	>128	0.0312	1	32	32
G2	Strong	16	>128	8	>128	≤0.0312	>64	2048	>64
G3	Moderate	2	8	4	>128	≤0.0312	0.5	16	8
G4	Moderate	2	>128	64	>128	≤0.0312	>64	2048	>64
G5	Moderate	8	>128	16	>128	≤0.0312	>64	2048	32
G6	Weak	0.25	>128	512	>128	≤0.0625	0.5	16	>64
G7	Weak	2	0.5	0.25	>128	≤0.0625	0.5	16	32
G8	Weak	8	>128	16	>128	0.5	0.5	1	32
G9	Weak	2	4	2	>128	0.125	0.5	4	32
G10	Weak	≤0.0625	≤0.125	2	>128	0.125	1	8	8
Standard strain^b^	Moderate	32	128	4	>128	≤0.0625	0.5	16	8

MIC breakpoints adapted and interpreted as sensitive, resistant, or intermediate as defined by CLSI criteria for metronidazole (sensitive: ≤8 *μ*g/mL; intermediate: = 16 *μ*g/mL; resistant: ≥32 *μ*g/mL) and clindamycin (sensitive: ≤2 *μ*g/mL; intermediate: = 4 *μ*g/mL; resistant: ≥8 *μ*g/mL) were used for interpreting MIC results; The ^a^MIC ratio was calculated using the following formula: MIC value with biofilm formation/MIC value without biofilm formation; ^b^*Gardnerella vaginalis* ATCC®14018 was used as a quality control bacterium and cultivated under anaerobic conditions.

## Data Availability

The data used to support the findings of this study are included within the article.

## Abbreviations

BV: Bacterial vaginosis
CDC: Centers for Disease Control and Prevention
BHI: Brain heart infusion
RFLP: Restriction fragment length polymorphism
MRS: De Man, Rogosa, and Sharpe
ODc: Cut-off OD
SD: Standard deviations
CLSI: Clinical and Laboratory Standards Institute
MIC: Minimum inhibitory concentration
MBEC: Minimum biofilm eradication concentration.
